# Self-Assembly CNTs@PANi Coffee Rings on Poly(styrene-ethylene-butylene-styrene) Triblock Copolymer for Largely Stretchable Electronics

**DOI:** 10.3390/polym12122847

**Published:** 2020-11-29

**Authors:** Ming Zhu, Ruifeng Zhang, Gang Chen, Wenjun He, Yaowei Chen, Deng-Guang Yu, Xiaoyan Li

**Affiliations:** School of Material Science and Engineering, University of Shanghai for Science and Technology, Shanghai 200093, China; spg960808@163.com (M.Z.); zrfzsh1314@163.com (R.Z.); ad18920315@163.com (G.C.); 13681620298@163.com (W.H.); c15825719366@163.com (Y.C.); ydg017@usst.edu.cn (D.-G.Y.)

**Keywords:** self-assembling, coffee ring, CNTs, PANi, stretchable, SEBS triblock copolymer

## Abstract

In this paper, CNTs@PANi nanocomposites were prepared by in-situ oxidation polymerization of aniline, and their structure, morphology and conductivity were characterized. A mixed solvent of toluene and tetrahydrofuran was used to prepare dispersions of CNTs@PANi and poly(styrene-ethylene-butylene-styrene) (SEBS) triblock copolymer, and bilayer composite film was prepared. According to the solvent phase separation and uneven evaporation flux, CNTs@PANi self-assembled into the interconnected coffee ring structure on the SEBS matrix. The prepared bilayer composite film had excellent stretchability, and the conductivity of the functional layer was close to that of CNTs@PANi, which could light up an LED lamp under 100% strain and restore the topological structure. Electrochemical tests showed that the bilayer film had obvious heterogeneity. The impedance characteristics of the CNTs@PANi functional layer and the SEBS matrix were analyzed, and its heterogeneous corrosion resistance mechanism further discussed.

## 1. Introduction

Largely stretchable electronic devices with sensitive responses have attracted great attention in various fields, such as wearable applications, electronic skins, flexible sensors, and supercapacitors [1,2,3,4]. Building a conductive network in a soft matrix is a common method for preparing stretchable electrons [5], but the risk of aggregation usually threatens the effectiveness of the conductive network, and the sensitivity is often reduced according to the encapsulation status in the soft matrix. By selectively combining planar rigid precursors on the stretched matrix, various mesoscopic wrinkle topologies can be realized on the top layer by strain release [6]. The wrinkle topology has good stretchability and is easy to combine with conventional electronic manufacturing methods [7,8]. However, the difficulty of the buckling strategy lies in the modulus mismatch between the rigid top layer and the soft matrix, and the wrinkle structure is unstable.

Solvent evaporation drive has been considered as an effective bottom-up self-assembly method [9]. During the solvent evaporation process, solute particles may usually flow through the capillary to the fixed contact line, forming coffee ring deposits [10,11,12]. The coffee ring effect can be used to assemble nanomaterials into patterned microstructures [13]. Shimoni et al. [14] performed inkjet printing of silver nanoparticles and formed a conductive ring pattern with a width of 5–10 μm through the coffee ring effect. Compared with the buckling strategy, the patterned microstructure of the coffee rings can form topological connections with a low threshold. More importantly, the gradient modulus of nanomaterials and soft substrates can also be established during the bottom-up self-assembly process, thus forming a transition between “hard” and “soft” like natural structural materials [15,16,17].

By selecting appropriate functional materials and a soft matrix to form composite materials with surface topology and hard–soft transition interfaces, we hope to obtain stretchable electrons with high sensitivity and large stretchability, and adapt to actual complex strain and environments. Traditional conductive metals are difficult to use to form long-term stable conformal interfaces due to their high modulus and severe mechanical mismatch with many soft tissues [18]. In this regard, various conductive functional materials have been extensively explored, including carbon nanotubes (CNTs) [19], conductive polymers [20], graphene [21], and metal nanowires [22].

CNTs are an ideal one-dimensional nanomaterial with low background current, wide potential window, stable electricity, and excellent mechanical properties [23]. At the same time, the active groups on CNTs can be used to bind biomolecules or chemical moieties for specific sensing and energy applications without compromising their inherent stretchability [24]. Unfortunately, due to the strong interaction between the nanotubes, their dispersibility in most solvents is hindered, making the direct processability of pristine CNTs always challenging [25]. Chemical surface grafting or in-situ polymerization can effectively improve the dispersibility of CNTs [26]. Aniline has a good affinity with CNTs, and in-situ polymerization can form π-π interaction between CNTs and polyaniline (PANi), which can improve electron mobility [27,28]. Encapsulating PANi on CNTs can reduce the aggregation of CNTs and improve their solvent processing performance, and has good mechanical properties and electrochemical stability [29]. Nanocomposites of CNTs and PANi can also form chemical interaction and physical entanglement with polymer chains, making a promising stretchable electronic functional material [30,31].

A stretchable matrix that can form a transition interface with functional materials is also very important. Poly(styrene-ethylene-butylene-styrene) (SEBS) is a thermoplastic elastomer (TPE) with hard polystyrene (PS) end blocks and soft poly (ethylene-butene (PEB)) mid-soft blocks [32,33,34,35,36]. Due to the thermodynamic incompatibility between covalently connected blocks, SEBS triblock copolymer has a variety of microphase separation morphology [37,38], with good stretchability and processability, almost no mechanical strain hysteresis under high frequency and large strain [39,40,41]. Bao’s group blended a conjugated polymer with SEBS and formed nano-constrained conjugated conductive nanofibers through phase separation, which showed good flexibility and high electron mobility, with no cracks under 100% tensile strain [42]. Compared with polydimethylsiloxane (PDMS) [43], SEBS is softer, has higher tensile properties, and can be processed repeatedly. More importantly, the microphase separation of SEBS triblock copolymer also helps to form compliant interfaces with various surface topologies, and can obtain soft–hard transition modulus during solvent evaporation.

In this paper, CNTs@PANi nanocomposites were prepared by aniline in-situ oxidative polymerization, and their structure, micro-morphology, and conductivity were characterized. A mixed solvent was used to prepare dispersions of CNTs@PANi and SEBS triblock copolymer, and bilayer composite films were prepared by spin coating. Driven by solvent phase separation and capillary evaporation, CNTs@PANi were assembled on the SEBS matrix into an interconnected coffee ring structure. The tensile properties and conductivity of the prepared bilayer film were tested and analyzed. Through impedance spectroscopy and Tafel polarization curve, the interface resistance of the CNTs@PANi functional layer and the SEBS matrix of the bilayer composite film were analyzed, and the heterogeneous electrochemical mechanism of the bilayer composite film was discussed.

## 2. Experimental

### 2.1. Materials

Linear triblock copolymer SEBS (M_w_ = 1.18 × 10^5^ g·mol^−1^) was obtained from Sigma-Aldrich Co. Ltd., Shanghai, China. Carbon nanotubes (CNTs, ≥95%) were purchased from Chinese Academy of Sciences Chengdu Organic Chemistry Co. Ltd., Chengdu, China. Hexadecyltrimethoxysilane (C_19_H_42_SiO_3_, M_w_ = 346.62 g·mol^−1^) and aniline monomer (≥99.5%) were obtained from Shanghai Aladdin Bio-Chem Technology Co. Ltd., Shanghai, China. A 304 stainless steel sheet was obtained from Baosteel Group Co. Ltd., Shanghai, China. Other analytical reagents, including sodium chloride (NaCl), ethanol, ammonium persulfate (APS, ≥98%), sodium dodecylbenzene sulfonate (SDS), hydrochloric acid (HCl, 36%–38%), toluene, and tetrahydrofuran (THF) were provided by Sinopharm Chemical Reagent Co. Ltd., Shanghai, China. All materials were used as received, and water was doubly distilled before use.

### 2.2. Preparation of CNTs@PANi

Before using CNTs, functionalized CNTs were obtained through the acidification method reported in our previous work [26]. We took a certain amount of functionalized CNTs aqueous dispersion in a flask, slowly added 1 mL of 1 M HCl dropwise, magnetically stirred for 10 min, ultrasounded for 10 min, added 0.3 mL of aniline monomer dropwise, magnetically stirred for 10 min, ultrasounded for 10 min to make it evenly dispersed, then weighed 183.8 mg of ammonium persulfate (APS) into 1 M HCl solution and sonicated for 5 min to fully dissolve. The APS solution was quickly poured into the above aniline and CNTs solution, magnetically stirred for 5 min, and allowed to stand at room temperature for 12 h. The above product was repeatedly washed with deionized water to neutrality by suction filtration, and then dispersed in water. The obtained product was named CNTs@PANi, and a part of it was freeze-dried for characterization.

### 2.3. Preparation of CNTs@PANi/SEBS Film

#### 2.3.1. PET Substrate Modification

The surface of the PET film was modified and used as a spin-coated substrate. We took 45 mL of ethanol and 5 mL water, added acetic acid to adjust the pH to 4~5, then added 1 mL of hexadecyltrimethoxysilane, and magnetically stirred for 1 h. The PET film was repeatedly washed with ethanol and acetone and dried, then immersed in the above solution for 10 min, taken out and dried naturally before use.

#### 2.3.2. Preparation of Spin Coating Solution

The CNTs@PANi aqueous dispersion was centrifuged at 6000 rpm to remove water, centrifuged twice with a mixed solvent of toluene and THF, then dispersed in the mixed solvent, and a small amount of SDS was added, then sonicated for 10 min. The volume ratio of toluene and THF was 6:4 (all mixed solvent in this ratio), and the solid content of CNTs@PANi was about 10 mg·mL^−1^. Taking 10 mL of CNTs@PANi mixed solvent dispersion, we added 0.5 g of SEBS, and magnetically stirred at room temperature for 6 h to obtain a functional spin coating solution. We then took 1 mL of CNTs@PANi mixed solvent dispersion, added 9 mL of the mixed solvent of toluene and THF, sonicated for 10 min, then added 1.5 g of SEBS, and magnetically stirred at room temperature for 6 h to obtain the base spin coating solution. For comparison, a functional spin coating solution with doubled CNTs@PANi concentration was also prepared. The monolayer composite film spin coating solution was prepared by the same method, in which 10 mL of CNTs@PANi mixed solvent dispersion was added with 1.5 g of SEBS. All spin coating solutions were used on the day they were prepared.

#### 2.3.3. Preparation of CNTs@PANi/SEBS Composite Film

**Figure 1:**
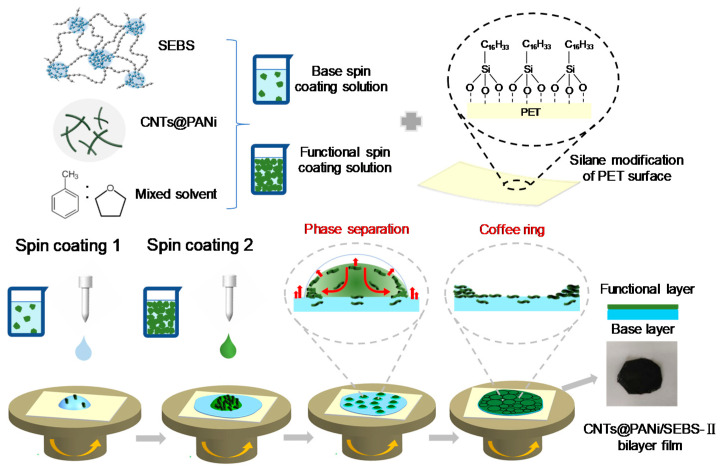
Schematic diagram of the preparation process of CNTs@PANi/SEBS-II bilayer film and the formation mechanism of coffee ring structure.

The schematic diagram of the preparation process is shown in Figure 1. Placing the modified PET substrate on the suction cup of the spin coater, we used a syringe to add 2 mL of the base spin coating solution, spinning at 600 rpm for 20 s, and then at 800 rpm for 30 s. We then immediately added 2 mL of the functional spin coating solution, spun it at 500 rpm for 25 s, removed it together with the PET substrate, and placed it in a fume hood to dry naturally for 3 h. After the solvent was completely evaporated, the edges were wetted with ethanol, and then the film was carefully peeled from the PET. The prepared sample was named CNTs@PANi/SEBS-II. A similar spin coating process was used to prepare monolayer composite film and SEBS film. The spin coating process was 600 rpm for 20 s and 800 rpm for 30 s, named as CNTs@PANi/SEBS-I.

### 2.4. Characterization

The chemical structures of CNTs@PANi were tested by FTIR (Spectrum 100 Perkin–Elmer, Billerica, MA, USA) and Raman spectroscopy with a laser excitation of 785 nm (400F Raman Spectrometer, Perkin–Elmer Co. Ltd., Billerica, MA, USA). UV–vis diffuse reflectance spectroscopy (Lambda 750, Perkin–Elmer Co., Ltd., Billerica, MA, USA) was used for optical absorption of PANi and CNTs@PANi, the solvent background was subtracted first and the scanning range was 220–800 nm. The morphology of the PANi, CNTs@PANi, and CNTs@PANi/SEBS films were observed by SEM (Quanta FEG450 FEI Co., Ltd., Hillsboro, OR, USA) at an accelerating voltage of 20 kV for PANi and 30 kV for the CNTs@PANi and film. The fracture cross section of the film was observed after tensile testing at room temperature or quenching in liquid nitrogen. The surface morphology of the film after tensile fracture was observed by Zeiss MERLIN Compact at 3 kV. All samples were sprayed with gold before the observation. The TEM was conducted on TECNAI F30 (FEI Corporation, Hillsboro, OR, USA) with an accelerating voltage of 300 kV. We dropped the diluted PANi or CNTs@PANi aqueous solution on the copper mesh and observed after natural drying.

A four-probe tester (Cascade M150, American Cascade Microtech, Beaverton, OR, USA) was used to measure the conductivity at room temperature. The freeze-dried CNTs@PANi powder was uniformly ground, and then pressed into a sheet with a diameter of 10 mm and a thickness of 0.55 mm. We cut the CNTs@PANi/SEBS film sample into a size of 10 × 20 mm^2^. During the measurement, the conductive silver glue was placed on the surface of the sample at an interval of 1 mm, and each group was tested 4 times and averaged. We used an LED lamp to show the conductivity of the film, clamped different sides of the film with conductive Fe sheet and insulating PET sheet, and then clamped them with a conductive alligator. A weight was hung to change the tensile strain and observe the change in the brightness of the LED lamp. A tensile testing machine (WDT-10) was used to test the tensile stress-strain curve of the film at room temperature with a gauge length of 5 mm and a tensile speed of 10 mm·min^−1^.

A multi-channel electrochemical test system (CHi660e, Shanghai Chenhua Instrument Co. Ltd., Shanghai, China) was used to measure the EIS test in a 3.5 wt% NaCl solution. We first measured the open circuit potential at a scan rate of 2 mV·s^−1^. Then, under the open circuit potential, we tested the impedance spectrum of the composite film; the frequency range was 1 Hz–100,000 Hz. We fitted the measured impedance data through Zview, making the error value less than 10%, and obtained the impedance value.

The Tafel curve was also measured in 3.5 wt% NaCl solution with a scan rate of 2 mV·s^−1^ from −1 V to +1 V. EIS and Tafel tests were used with the classic three-electrode configuration. For the Tafel test, we cut the 304SS sheet (1 mm thick) into 15 × 13 mm^2^ pieces, washed with ethanol and dried before use. The metal wire was sealed on one side of the steel sheet with paraffin wax, and paraffin was further applied until the exposed steel sheet was 10 × 10 mm^2^, which was used as a working electrode. The film sample was attached to the 304SS sheet as working electrode (the functional layer and the base layer of the bilayer film were connected to the 304SS electrode respectively). Similarly, the film sample was attached to the Indium tin oxide (ITO) conductive glass as the working electrode for EIS measurement. The calomel electrode was used as reference electrode, and a platinum foil of 10 × 10 mm^2^ was used as the counter electrode. All experiments were performed at room temperature.

## 3. Results and Discussion

### 3.1. Structure and Morphology of CNTs@PANi

**Figure 2:**
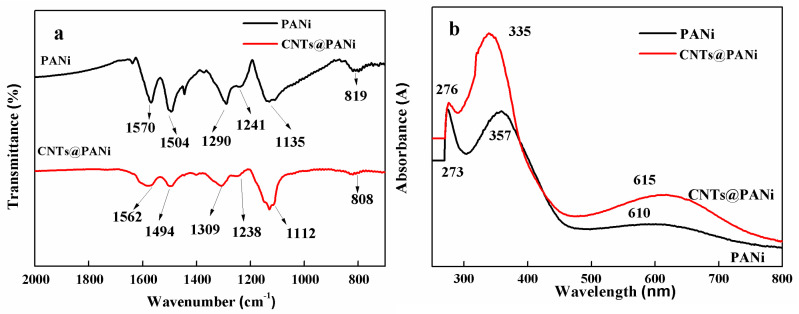

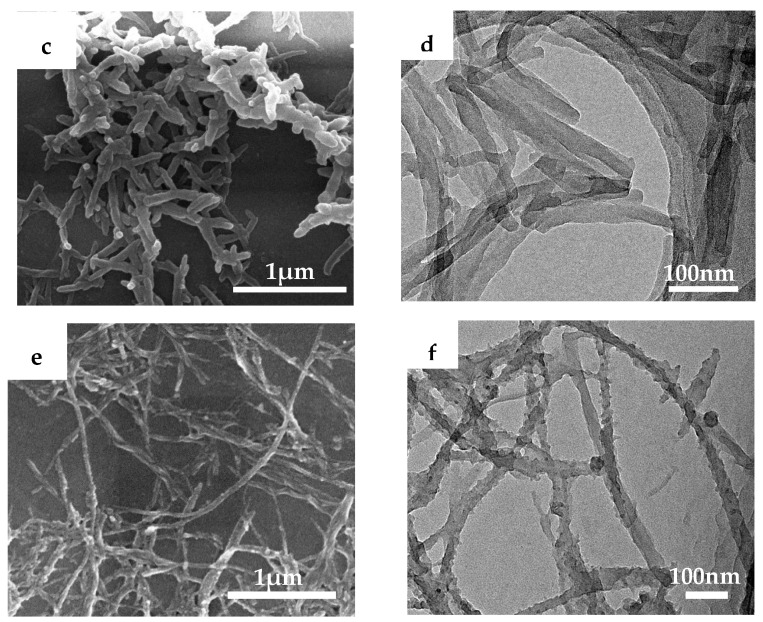
The spectra and morphology of PANi and CNTs@PANi: (**a**) FTIR; (**b**) UV–vis; (**c**) SEM image of PANi; (**d**) TEM image of PANi; (**e**) SEM image of CNTs@PANi; (**f**) TEM image of CNTs@PANi.

As can be seen from FTIR spectrum (Figure 2a), PANi has characteristic peaks at 1570, 1504, 1290, 1241, 1135, and 819 cm^−1^, which correspond to the quinoid vibration, benzene vibration, and aromatic amine C–N vibration of PANi, –N=Q=N– vibration, C-H bending vibrations in and out of the benzene ring, respectively. CNTs@PANi contains all the characteristic peaks of PANi, in which the absorption peaks of the quinone structure and the benzene structure are red-shifted to 1562 cm^−1^ and 1494 cm^−1^, and the peak ratio of the quinone/benzene increases, indicating that there is π-π conjugation between the in-situ polymerized PANi and CNTs, and it mainly occurred on the quinone ring of PANi. In the UV spectrum (Figure 2b), the peaks at 273 and 357 nm are π-π electronic transition absorption, of which 273 nm corresponds to PANi oligomers [44]. The peak at 610 nm corresponds to the n-π of the anthracene ring structure. The π-π transition blue shifted to 335 nm in CNTs@PANi, and the n-π transition red shifted to 615 nm, indicating that conjugated interactions with CNTs occurred during the in-situ polymerization of PANi, which is consistent with the results of FTIR.

The prepared PANi (Figure 2c,d) had a smooth surface and short nanofiber morphology with diameter of 40–50 nm and length of about 300–400 nm, while the surface of CNTs@PANi (Figure 2e,f) was rough. The surface of acidified CNTs contain functional groups such as –COOH, which can adsorb aniline monomers through electrostatic interaction, and then the in-situ polymerization reaction on the tube wall makes PANi finally encapsulated on the CNTs.

### 3.2. Morphology of CNTs@PANi/SEBS Film

The choice of solvent is very important for the preparation of composite films. Due to the hydrogen bond interaction between tetrahydrofuran (THF) and water [45], CNTs@PANi can be transferred from the aqueous dispersion to the THF solvent. According to our previous research [46], the solubility parameter of PANi is closer to THF and is more likely to be dispersed in THF (Figure S1), while THF and toluene are mutually soluble and both are good solvents for SEBS (Table S1). Here, the mixed solvent of THF and toluene was applied to prepare composite films.

**Figure 3:**
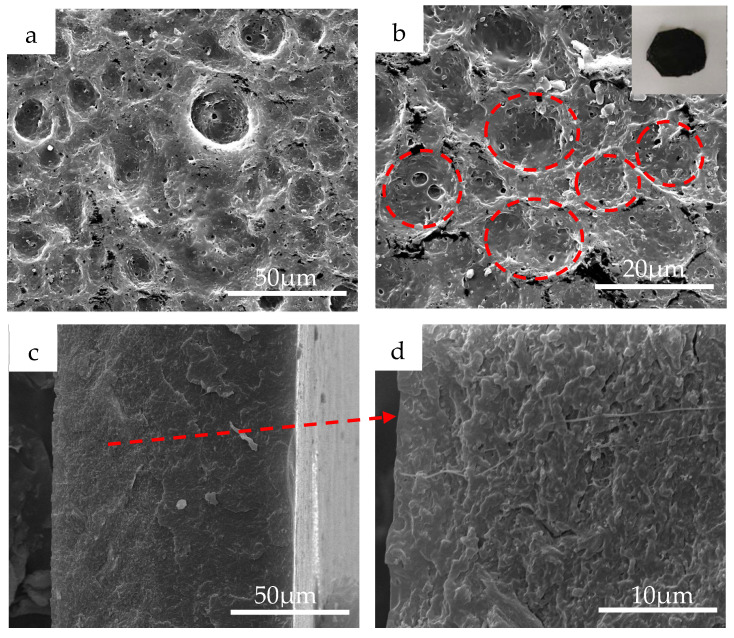
SEM images of CNTs@PANi/SEBS-II: (**a**) surface; (**b**) enlarged surface; (**c**) quenched section; (**d**) enlarged view of (**c**).

As shown in Figure 3a,b, the surface morphology of the bilayer film showed a micron-level interconnected coffee ring topology. After spin coating the SEBS base layer, we immediately spin coated the CNTs@PANi functional solution. Since CNTs@PANi tends to be dispersed in THF, CNTs@PANi-rich droplets formed on the SEBS base layer due to solvent phase separation. The boiling point of THF is lower than that of toluene, and the saturated vapor pressure is higher (Table S2). When low-boiling THF preferentially evaporated, CNTs@PANi flowed with the capillary fluid to compensate for the evaporation loss at the edge of the gas-liquid interface and accumulated on the edge of the droplets. The outer layer of the droplets shrank under the action of surface tension, and the CNTs@PANi approached each other and formed a coffee ring structure (Figure 1). The surface morphology of CNTs@PANi/SEBS-Ι monolayer film was completely different, showing micron-sized pores (Figure S2a). This is due to the overall solvent phase separation in the monolayer film. With the preferential volatilization of THF, CNTs@PANi will eventually remain around the pores left by THF.

The functional spin-coating solution in the above bilayer film with the coffee ring topology contained about 1 wt% of CNTs@PANi (the content of CNTs@PANi in the basic spin-coating solution is less than 0.1 wt%). When the content of CNTs@PANi in the functional spin coating solution is too high (2 wt%), as the solvent evaporates, the excess CNTs@PANi will hinder the collapse of the droplets, thus failing to form a recessed coffee ring, showing a relatively flat surface morphology (Figure S3).

Unlike the agglomeration phenomenon in the quenched cross section of the monolayer film (Figure S2b), CNTs@PANi are uniformly enriched in the top of the bilayer film, without obvious interface delamination (Figure 3d). The top spin coating solution has certain wettability with the SEBS layer that has not yet been completely dried. As the solvent evaporates slowly throughout the film, the SEBS molecular chains will migrate and entangle together, and gradually adapt to the difference through the microphase separation of the PS hard segment and the PEB soft segment, and finally form a soft–hard transition interface.

### 3.3. Conductivity and Stretchability of CNTs@PANi/SEBS Film

The π-π interaction between PANi and CNTs can increase carrier delocalization and conductivity (Figure S4). The conductivity of PANi fiber is about 1.6 × 10^−3^ S·cm^−1^. When the CNTs content is about 12 wt%, the conductivity of CNTs@PANi increases to about 4.1 × 10^−1^ ± 0.1 S·cm^−1^. All composite films in this article used the above CNTs@PANi with 12 wt% CNTs content. The conductivity of the CNTs@PANi/SEBS-II functional layer with coffee ring topology was about 3.4 × 10^−1^ ± 0.1 S·cm^−1^ (SEBS base layer was about 1.4 × 10^−5^ S·cm^−1^), which was close to the CNTs@PANi.

Additionally, for the bilayer film with flat surface morphology, although the content of CNTs@PANi in the functional layer doubled, the corresponding conductivity was only about 8.9 × 10^−2^ S·cm^−1^. The Raman test (Figure S5) showed that only the G peak of CNTs (1589 cm^−1^) in the monolayer film was obvious, while the top of the bilayer film not only had a higher G peak, but also obvious PANi peaks (consistent with the peak of PANi in the inset of Figure S5). This indicates that the CNTs@PANi coffee ring topology was assembled on the surface of the SEBS matrix, rather than encapsulated in the SEBS, which is very important for effective conductive paths.

**Figure 4:**
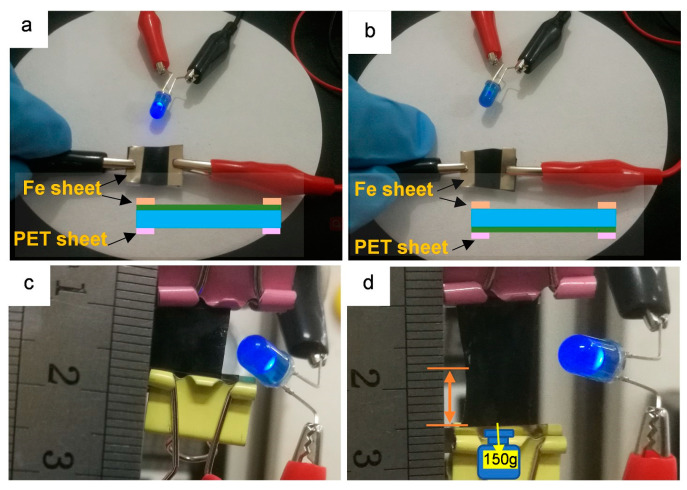
LED conductivity test of CNTs@PANi/SEBS-II bilayer film: (**a**) functional layer contact with Fe sheet; (**b**) SEBS base layer contact with Fe sheet; (**c**) unstretched initial state; (**d**) about 100% strain in the stretched state.

According to reports, in the CNTs and SEBS composites prepared by solvent blending, the conductivity is 1 × 10^−2^ S·cm^−1^ when the CNT content is 4 wt% [41], while the penetration threshold of PANi for PANi/SEBS composites is up to 10 wt% [47]. In this work, CNTs@PANi/SEBS-II containing only 1 wt% CNTs@PANi obtained good conductivity, as shown in Figure 4. After attaching the CNTs@PANi/SEBS-II functional layer to the conductive iron sheet, the LED light could be lit (Figure 4a), but the SEBS base layer could not (Figure 4b), and neither could the monolayer film. Compared with the initial state (Figure 4c), CNTs@PANi/SEBS-II functional layer still lit up the LED lamp under 100% strain (Figure 4d), which indicates that the coffee ring topology still maintained conductive paths under large strain.

**Figure 5:**
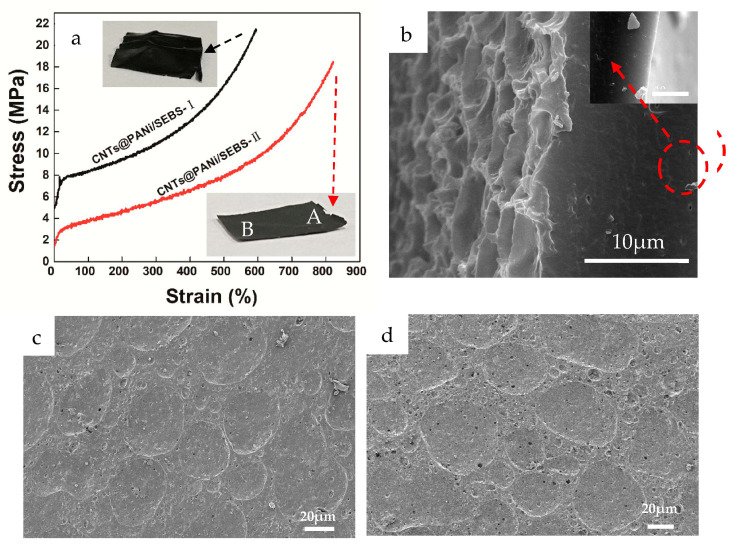
(**a**) Tensile stress-strain curves, with digital film photos after fracture; SEM images of CNTs@PANi/SEBS-II bilayer film for (**b**) tensile fractured cross-sections; (**c**) surface after tensile fracture, area A; (**d**) surface after tensile fracture, area B.

Both monolayer and bilayer composite films showed excellent stretchability (Figures S6 and S7), and their tensile stress-strain curves and tensile section morphology are shown in Figure 5. The tensile strength, elongation at break and elastic modulus of the bilayer film were 18.5 MPa, 821%, and 1.0 MPa (the monolayer film was 21.5 MPa, 595%, and 19.5 MPa, respectively), showing good strength, large strain and low elastic modulus, which can match human skin tissues.

Compared with the rougher tensile fracture cross section of the monolayer film (Figure S8), the tensile cross section of the bilayer film was relatively smooth (Figure 5b), and there was no obvious interface delamination between the functional layer and the base layer, showing a gradient and uniform stress transfer. It is worth noting that there were some circular holes (<1 um) near the base in the tensile section. This is because there were some invisible defects in the composite film. When subjected to a large tensile strain, these micro-defects will deform and elongate, and as the elasticity of SEBS recovers, these micro-defects will return to the round hole. SEBS can effectively prevent crack propagation. Interestingly, there were fewer pores in the functional layer, which may be because the solvent in the functional layer had completely evaporated and defects were less likely to occur.

After tensile fracture, the bilayer film was relatively flat compared to the monolayer film (Figure 5a insets), with only slight wrinkles near the fracture. On the surface of the bilayer film, the coffee ring structures near (Figure 5c, corresponding area A) and far from the fracture (Figure 5d, corresponding area B) still existed and remained connected to each other, but some micropores appeared. After stretching and breaking, the recovered bilayer film still lit up the LED lamp along the stretching direction (Figure S9a) and perpendicular to the stretching direction (Figure S9b). The coffee ring topology showed a large strain recoverable conductivity.

Generally, the mechanical hysteresis of elastomer composites containing CNTs was increased compared to the original polymer [48]. Fortunately, compared with the monolayer film, the prepared bilayer film had lower modulus and higher elongation at break. It is foreseeable that the hysteresis loss of the bilayer film with CNTs@PANi coffee ring topology will be lower than that of the monolayer film.

### 3.4. Electrochemical Properties of CNTs@PANi/SEBS Film

**Figure 6:**
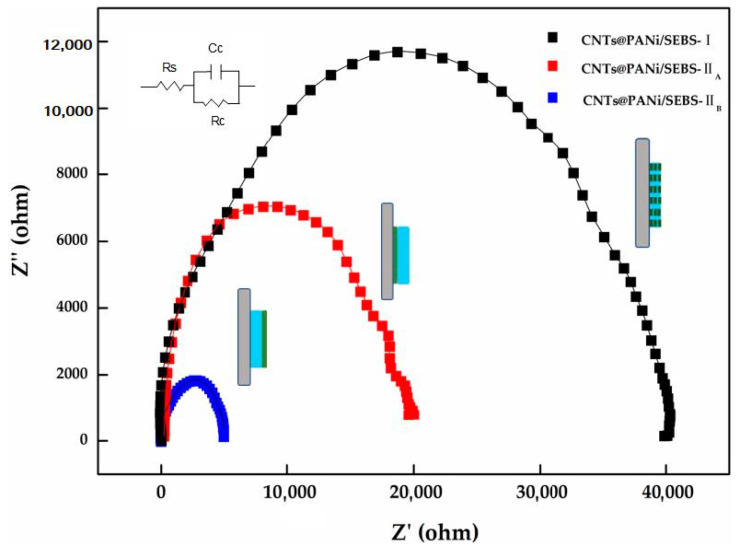
Electrochemical impedance curves of CNTs@PANi/SEBS composite films.

Electrochemical impedance spectroscopy (EIS) was used to study the electrochemical performance of the composite film, and a Nyquist diagram was obtained, as shown in Figure 6. Table 1 lists the electrochemical parameters corresponding to the equivalent circuit, where R_s_ is the solution resistance, and C_c_ and R_c_ are the capacitance and resistance of the film. According to Nyquist results, the monolayer film had the highest impedance. Interestingly, when the CNTs@PANi layer was attached to the ITO electrode (CNTs@PANi/SEBS-II_A_), R_c_ and C_c_ were 18,080 Ω·cm^2^ and 1.36 × 10^−8^ F·cm^−2^. When the SEBS layer was attached to the electrode (CNTs@PANi/SEBS-II_B_), R_c_ and C_c_ were 4080 Ω·cm^2^ and 3.41 × 10^−7^ F·cm^−2^, respectively. The above results indicate that when the CNTs@PANi is in contact with the electrolyte, its electrochemical activity increases, while when the SEBS matrix layer is in contact with the electrolyte, its electrochemical activity is greatly reduced, and the bilayer film shows obvious heterogeneity.

**Figure 7:**
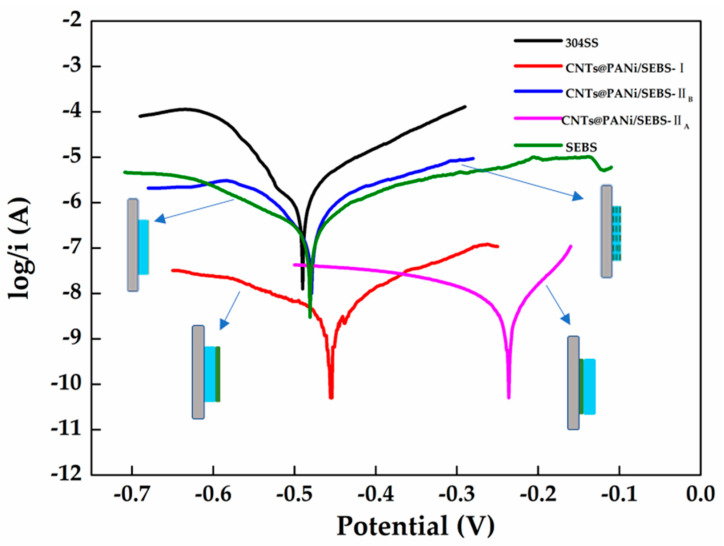
Tafel curves of CNTs@PANi/SEBS composite films.

The Tafel polarization curve is shown in Figure 7, and the Tafel polarization parameters are shown in Table 2. The corrosion potential (E_corr_) of SEBS spin-coated film was similar to that of 304SS, with corrosion current (I_corr_) of 5.374 × 10^−7^ A·cm^−2^, corrosion resistance (R_p_) of 54,409 Ω·cm^2^, and corrosion rate (V_corr_) of 0.0060 mm/year (V_corr_ of 304SS is 0.0270 mm/year). The SEBS film had a certain barrier protection effect on 304SS, but the soft segment of PEB in the SEBS triblock copolymer had obvious relative thermal movement, and the chloride ions in the electrolyte could penetrate the SEBS and corrode iron.

It is worth noting that when the functional layer of the bilayer film was in contact with 304SS (CNTs@PANi/SEBS-II_A_), its E_corr_ rose to −0.237 V and V_corr_ was 0.0003 mm/year. The cathodic polarization curve was smoother, and the corrosion changed from cathodic control process to anode control, showing the best anti-corrosion effect. The oxidation potential of PANi was higher than that of iron, and the oxidation-reduction potential was lower than that of oxygen, and PANi could be repeatedly switched and oxidized between the intrinsic state and the reduced state. The CNTs@PANi in contact with the stainless-steel surface transferred the electrons lost due to the iron anode reaction to PANi. Fe^2+^ further lost electrons at the interface with CNTs@PANi, forming a passivation film, which is consistent with our previous research [46]. CNTs@PANi had good electron transport ability, so the reduction reaction of cathode oxygen occurred in the CNTs@PANi functional layer, and then diffused the generated -OH into the electrolyte. On the other hand, due to the barrier effect of the SEBS polymer layer, it also shielded the oxygen and chloride ions to a certain extent, thereby protecting the passivation layer of 304SS from damage. Interestingly, the surface topology also affected Tafel’s results. For the bilayer film with the flat morphology (Figure S10), when the functional layer was in contact with 304SS, although the content of CNTs@PANi was higher, E_corr_ increased to 0.0340 V, but R_p_ was 455,795 Ω·cm^2^ and V_corr_ was 0.0008 mm/year, not as good as the monolayer film (0.0006 mm/year). In this flat surface morphology, the SEBS molecular chain covered CNTs@PANi functional material, thereby affecting the passivation protection effect.

When the functional layer was in contact with the electrolyte (CNTs@PANi/SEBS-II_B_), CNTs@PANi tended to adsorb chloride ions, resulting in a potential difference at the electrolyte interface and lower surface resistance. In addition, the internal non-polar SEBS layer delayed the charging and discharging process of CNTs@PANi, thereby increasing the capacitance value and also making corrosion easier. The same was true for the bilayer film with the flat surface morphology (Figure S11). Its E_corr_ was −0.52 V, R_p_ was 9007 Ω·cm^2^, and V_corr_ was 0.0380 mm/year, which intensified the corrosion of 304SS.

## 4. Conclusions

In this paper, CNTs@PANi conductive nanocomposites were prepared by in-situ oxidation polymerization of aniline. The mixed solvent of toluene and THF was used to prepare dispersions of CNTs@PANi and SEBS triblock copolymer, and bilayer composite films were prepared by spin coating. Due to solvent phase separation and uneven evaporation flux, CNTs@PANi self-assembled into the coffee ring topology on the surface of SEBS; the corresponding conductivity was close to that of CNTs@PANi, and could illuminate the LED lamp under 100% strain. The elastic modulus of the prepared CNTs@PANi/SEBS-II was only 1.0 MPa, while the tensile strength reached 18.5 MPa. The elongation at break of bilayer film was as high as 821%, and the coffee ring topology could still be recovered after breaking, showing considerable strain recovery ability. EIS showed that when the CNTs@PANi functional side was in contact with the electrolyte, ions were easily adsorbed to the surface of the composite film, showing lower surface resistance and larger capacitance. When the SEBS matrix was in contact with the electrolyte, it had a larger resistance and smaller capacitance. The Tafel test showed that when the CNTs@PANi topology was attached to 304SS, significant passivation occurred, which can greatly improve the corrosion resistance of stainless steel. This heterogeneous composite film with multiple properties can enrich the applications of stretchable electronics. The self-assembly method proposed in this paper can reduce the dependence on the chemical and physical uniformity of the substrate, and is expected to be applied to other flexible and stretchable functional composite materials.

## Figures and Tables

**Table 1:** Impedance parameters of CNTs@PANi/SEBS composite films.

Sample	R_s_ (Ω·cm^2^)	R_c_ (Ω·cm^2^)	C_c_ (F·cm^−2^)
CNTs@PANi/SEBS-I	3810	32,083	6.93 × 10^−8^
CNTs@PANi/SEBS-II_A_	241	18,080	1.36 × 10^−8^
CNTs@PANi/SEBS-II_B_	160	4080	3.41 × 10^−7^

**Table 2:** Tafel polarization parameters of CNTs@PANi/SEBS composite films.

Sample	E_corr_ (V)	I_corr_ (A·cm^−2^)	R_p_ (Ω·cm^2^)	V_corr_ (mm/year)
304SS	−0.490	1.943 × 10^−6^	9798	0.0270
SEBS	−0.481	5.374 × 10^−7^	54,409	0.0060
CNTs@PANi/SEBS-I	−0.456	5.445 × 10^−9^	5,400,049	0.0006
CNTs@PANi/SEBS-II_A_	−0.237	3.760 × 10^−9^	3,252,258	0.0003
CNTs@PANi/SEBS-II_B_	−0.481	9.753 × 10^−7^	46,119	0.0012

## Supplementary Materials

The following are available online at https://www.mdpi.com/2073-4360/12/12/2847/s1. Figures S1−S11: PANi dispersion status in THF and toluene, SEM image of surface and quenched cross section of CNTs@PANi/SEBS-Ι monolayer film, surface SEM image of the bilayer film with doubled CNTs@PANi content in the functional layer, SEM image the bilayer film and the cross section of bilayer film conductivity of CNTs@PANi with different CNTs content, Raman spectra of CNTs@PANi, monolayer film and function layer of CNTs@PANi/SEBS-II bilayer film, stretchable photos of CNTs@PANi/SEBS-Ι monolayer film and CNTs@PANi/SEBS-II bilayer film, SEM image of tensile fractured cross section of CNTs@PANi/SEBS-Ι monolayer film, digital photos of CNTs@PANi/SEBS-Ι and CNTs@PANi/SEBS-II composite film after tensile fracture, surface SEM of close to the fracture and away from fracture of bilayer film after tensile fracture, LED conductivity test of bilayer film after tensile fracture of along and perpendicular to the stretching direction, the impedance curves and Tafel curves of the bilayer film with flat surface morphology (doubled CNTs@PANi content in the functional layer). Tables S1−S2: Hildebrand and Hansen’s solubility parameters, physical parameters of different solvents and the Flory–Huggins interaction parameter χ.

## References

[1] Zeng J., Dong L., Sha W., Wei L., Guo X. (2020). Highly stretchable, compressible and arbitrarily deformable all-hydrogel soft supercapacitors. Chem. Eng. J..

[2] Yang Y., Sun N., Wen Z., Cheng P., Zheng H., Shao Y., Xia Y., Chen C., Lan H., Xie X. (2018). Liquid-metal-based super-stretchable and structure-designable triboelectric nanogenerator for wearable electronics. ACS Nano.

[3] Liu Y., He K., Chen G., Leow W., Chen X. (2017). Nature-inspired structural materials for flexible electronic devices. Chem. Rev..

[4] Son D., Bao Z. (2018). Nanomaterials in skin-inspired electronics: Toward soft and robust skin-like electronic nanosystems. ACS Nano.

[5] Jiang S., Zhang H., Song S., Ma Y., Li J., Lee G., Han Q., Liu L. (2015). Highly stretchable conductive fibers from few-walled carbon nanotubes coated on poly(m-phenylene isophthalamide) polymer core/shell structures. ACS Nano.

[6] Liu Z., Qi D., Guo P., Liu Y., Zhu B., Yang H., Liu Y., Li B., Zhang C., Yu J. (2015). Thickness-gradient films for high gauge-factor stretchable strain sensors. Adv. Mater..

[7] Wu X., Han Y., Zhang X., Lu C. (2017). Spirally structured conductive composites for highly stretchable, robust conductors and sensors. ACS Appl. Mater. Interfaces.

[8] Hu X., Dou Y., Li J., Liu Z. (2019). Buckled structures: Fabrication and applications in wearable electronics. Small.

[9] Al-Milaji K., Zhao H. (2019). New Perspective of mitigating the coffee-ring effect: Interfacial assembly. J. Phys. Chem. C.

[10] Zhang Z., Zhang X., Xin Z., Deng M., Wen Y., Song Y. (2013). Controlled inkjetting of a conductive pattern of silver nanoparticles based on the coffee—ring effect. Adv. Mater..

[11] Choi S., Stassi S., Pisano A.P., Zohdi T.I. (2010). Coffee-ring effect-based three dimensional patterning of micro/nanoparticle assembly with a single droplet. Langmuir.

[12] Mayarani M., Basavaraj M.G., Satapathy D.K. (2018). Viscoelastic particle–laden interface inhibits coffee-ring formation. Langmuir.

[13] Goh G.L., Saengchairat N., Agarwala S., Yeong W.Y., Tran T. (2019). Sessile droplets containing carbon nanotubes: A study of evaporation dynamics and CNT alignment for printed electronics. Nanoscale.

[14] Shimoni A., Azoubel S., Magdassi S. (2014). Inkjet printing of flexible high-performance carbon nanotube transparent conductive films by “coffee ring effect”. Nanoscale.

[15] Chen K., Zhang S., Li A., Tang X., Li L., Guo L. (2018). Bioinspired interfacial chelating-like reinforcement strategy toward mechanically enhanced lamellar materials. ACS Nano.

[16] Zhong J., Ma Y., Song Y., Zhong Q., Chu Y., Karakurt I., Bogy D.B., Lin L. (2019). A flexible piezoelectret actuator/sensor patch for mechanical human–machine interfaces. ACS Nano.

[17] Yin B., Wen Y., Hong T., Xie Z., Yuan G., Ji Q., Jia H. (2017). Highly stretchable, ultrasensitive, and wearable strain sensors based on facilely prepared reduced graphene oxide woven fabrics in an ethanol flame. ACS Appl. Mater. Interfaces.

[18] Choi S., Han S., Kim D., Hyeon T., Kim D.H. (2019). High-performance stretchable conductive nanocomposites: Materials, processes, and device applications. Chem. Soc. Rev..

[19] Moon G., Joo J., Yin Y. (2013). Stacked multilayers of alternating reduced graphene oxide and carbon nanotubes for planar supercapacitors. Nanoscale.

[20] Wang X.X., Yu G.F., Zhang J., Yu M., Ramakrishna S., Long Y.Z. (2021). Conductive polymer ultrafine fibers via electrospinning: Preparation, physical properties and applications. Prog. Mater. Sci..

[21] Sun Z., Chang H. (2014). Graphene and graphene-like two-dimensional materials in photodetection: Mechanisms and methodology. ACS Nano.

[22] Xu J., Wang K., Li Y., Zhuang T.T., Gao H.L., Liu Y.Y., He C.X., Yu S.H. (2020). Regulating silver nanowire size enables efficient photoelectric conversion. Sci. China Chem..

[23] Cha C., Shin S., Annabi N., Dokmeci M.R., Khademhosseini A. (2013). Carbon-based nanomaterials: Multifunctional materials for biomedical engineering. ACS Nano.

[24] Zhu L., Zhou X., Liu Y., Fu Q. (2019). Highly sensitive, ultrastretchable strain sensors prepared by pumping hybrid fillers of carbon nanotubes/cellulose nanocrystal into electrospun polyurethane membranes. ACS Appl. Mater. Interfaces.

[25] Yang Z., Cao Z., Sun H., Li Y. (2008). Composite films based on aligned carbon nanotube arrays and a poly(n−isopropyl acrylamide) hydrogel. Adv. Mater..

[26] Vankalaya R., Lai W.J., Cheng K.C., Hwang K. (2011). Enhanced electrical conductivity of nylon 6 composite using polyaniline-coated multi-walled carbon nanotubes as additives. Polymer.

[27] Kumar S., Saeed G., Zhu L., Hui K., Kim N., Lee J. (2021). 0D to 3D carbon-based networks combined with pseudocapacitive electrode material for high energy density supercapacitor: A review. Chem. Eng. J..

[28] Wang H., Meng F., Huang F., Jing C., Li Y., Wei W., Zhou Z. (2019). Interface modulating CNTs@PANi hybrids by controlled unzipping of the walls of CNTs to achieve tunable high-performance microwave absorption. ACS Appl. Mater. Interfaces.

[29] Hu R., Wang Y., Zhao J., Jiang R., Zheng J. (2019). Fabrication of stretchable multi-element composite for flexible solid-state electrochemical capacitor application. Chem. Eng. J..

[30] Zhang J., Li X., Wang X., Qiu B., Li Z., Ding J. (2016). Preparation of vertically aligned carbon nanotube/polyaniline composite membranes and the flash welding effect on their supercapacitor properties. RSC Adv..

[31] Xu J., Ding J., Zhou X., Zhang Y., Zhu W., Liu Z., Ge S., Yuan N., Fang X., Baughman R.H. (2017). Enhanced rate performance of flexible and stretchable linear supercapacitors based on polyaniline@Au@carbon nanotube with ultrafast axial electron transport. J. Power Sources.

[32] Li J., Li X., Wei Q., Yang J., Qiu B., Xu J., Wang X. (2018). Synergistic effect of organophosphate functionalized montmorillonite on properties and water resistance of intumescent flame—retarded SEBS. Fire Mater..

[33] Castañeda S., Ribadeneira R. (2020). Description of hydroxide ion structural diffusion in a quaternized SEBS anion exchange membrane using Ab initio molecular dynamics. J. Phys. Chem. C.

[34] Li X., Yu H., Kang X., Chen G., Zhu M., Xu J. (2020). Effect of injection molding on structure and properties of poly(styrene—ethylene—butylene—styrene) and its nanocomposite with functionalized montmorillonite. J. Appl. Polym. Sci..

[35] Yu H., Li J., Chen G., Zhang R., Li Y., Qiu B., Li X. (2020). Effects of phosphate emulsion—based montmorillonite on structure and properties of poly(styrene-ethylene-butylene-styrene) triblock copolymer. Polym. Eng. Sci..

[36] Li X., Yang J., Zhou X., Wei Q., Li J., Qi B., Wunderlich K., Wang X. (2018). Effect of compatibilizer on morphology, rheology and properties of SEBS/clay nanocomposites. Polym. Test..

[37] Pantoja M., Jian P.Z., Cakmak M., Cavicchi K.A. (2019). Shape memory properties of polystyrene-block-poly(ethylene-co-butylene)-block-polystyrene (SEBS) ABA triblock copolymer thermoplastic elastomers. ACS Appl. Polym. Mater..

[38] Gennari C., Quaroni G., Creton C., Minghetti P., Cilurzo F. (2020). SEBS block copolymers as novel materials to design transdermal patches. Int. J. Pharm..

[39] Kurusu R.S., Demarquette N.R. (2016). Wetting of hydrophilic electrospun mats produced by blending SEBS with PEO–PPO–PEO copolymers of different molecular weight. Langmuir.

[40] Krishnan S., Ward R.J., Hexemer A., Sohn K.E., Lee K.L., Angert E.R., Fischer D.R., Kramer E.J., Ober C.K. (2006). Surfaces of fluorinated pyridinium block copolymers with enhanced antibacterial activity. Langmuir.

[41] Yang X., Sun L., Zhang C., Huang B., Chu Y., Zhao B. (2019). Modulating the sensing behaviors of poly(styrene-ethylene-butylenestyrene)/carbon nanotubes with low-dimensional fillers for large deformation sensors. Compos. Part B.

[42] Xu J., Wang S., Wang G.J.N., Zhu C., Luo S., Jin L., Gu X., Chen S., Feig V.R., To J.W. (2017). Highly stretchable polymer semiconductor films through the nanoconfinement effect. Science.

[43] Goel P., Kumar S., Sarkar J., Singh J.P. (2015). Mechanical strain induced tunable anisotropic wetting on buckled PDMS silver nanorods arrays. ACS Appl. Mater. Interfaces.

[44] Chen I.W., Chou Y.C., Wang P.Y. (2019). Integration of ultrathin MoS2/PANI/CNT composite paper in producing All-Solid-State flexible supercapacitors with exceptional volumetric energy density. J. Phys. Chem. C.

[45] Purkayastha D., Madhurima V. (2013). Interactions in water–THF binary mixture by contact angle, FTIR and dielectric studies. J. Mol. Liq..

[46] Zhang R., Huang K., Zhu M., Chen G., Tang Z., Li Y., Yu H., Qiu B., Li X. (2019). Corrosion resistance of stretchable electrospun SEBS/PANi micro-nano fiber membrane. Eur. Polym. J..

[47] Costa P., Oliveira J., Horta-Romarís L., Abad M.-J., Moreira J., Zapiráin I., Aguado M., Galván S., Lanceros-Mendez S. (2018). Piezoresistive polymer blends for electromechanical sensor applications. Compos. Sci. Technol..

[48] Ribeiro S., Costa P., Ribeiro C., Sencadas V., Botelho G., Lanceros-Méndez S. (2014). Electrospun styrene–butadiene–styrene elastomer copolymers for tissue engineering applications: Effect of butadiene/styrene ratio, block structure, hydrogenation and carbon nanotube loading on physical properties and cytotoxicity. Compos. Part B Eng..

