# Supplementary material for: Self-Assembly CNTs@PANi Coffee Rings on Poly(styrene-ethylene-butylene-styrene) Triblock Copolymer for Largely Stretchable Electronics
Source: Polymers (Basel). 2020 Nov 29;12(12):2847. doi: 10.3390/polym12122847 (PMC7760645; doi:10.3390/polym12122847)
Supplement: Supplementary file 1 [file polymers-12-02847-s001.pdf]

Supplementary Material

Self-assembly CNTs@PANi coffee rings on poly(styrene-ethylene-butylene-styrene) triblock copolymer for largely stretchable electronics

Ming Zhu, Ruifeng Zhang, Gang Chen, Wenjun He, Yaowei Chen, Deng-guang Yu, Xiaoyan Li*

School of Material Science and Engineering, University of Shanghai for Science and Technology, Shanghai 200093, China

E-mail: lixiaoyan@usst.edu.cn

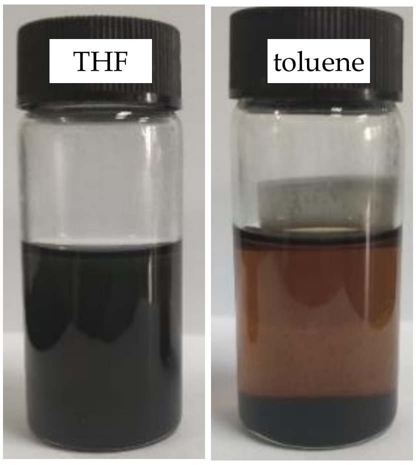

Fig. S1. PANi dispersion status in THF and toluene.

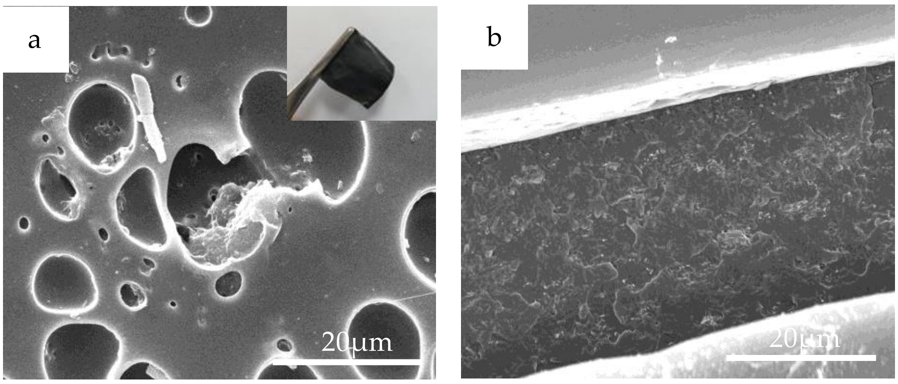

Fig. S2. SEM images of CNTs@PANi/SEBS-I (a) surface; (b) quenched cross section.

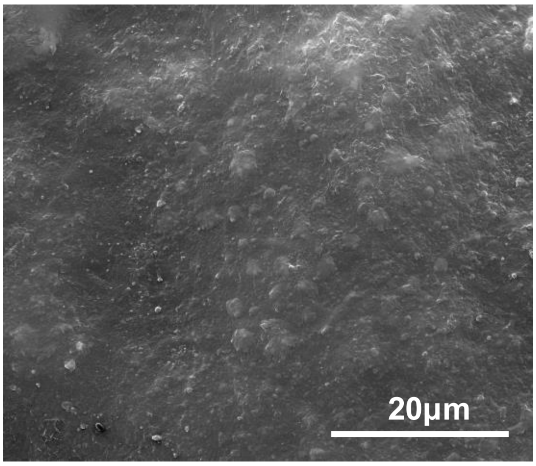

Fig. S3. Surface SEM image of the bilayer film with doubled CNTs@PANi content in the functional layer.

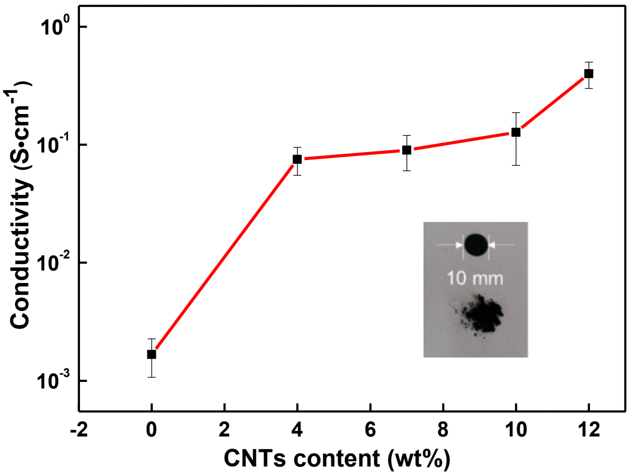

Fig. S4. The conductivity of CNTs@PANi with different CNTs content.

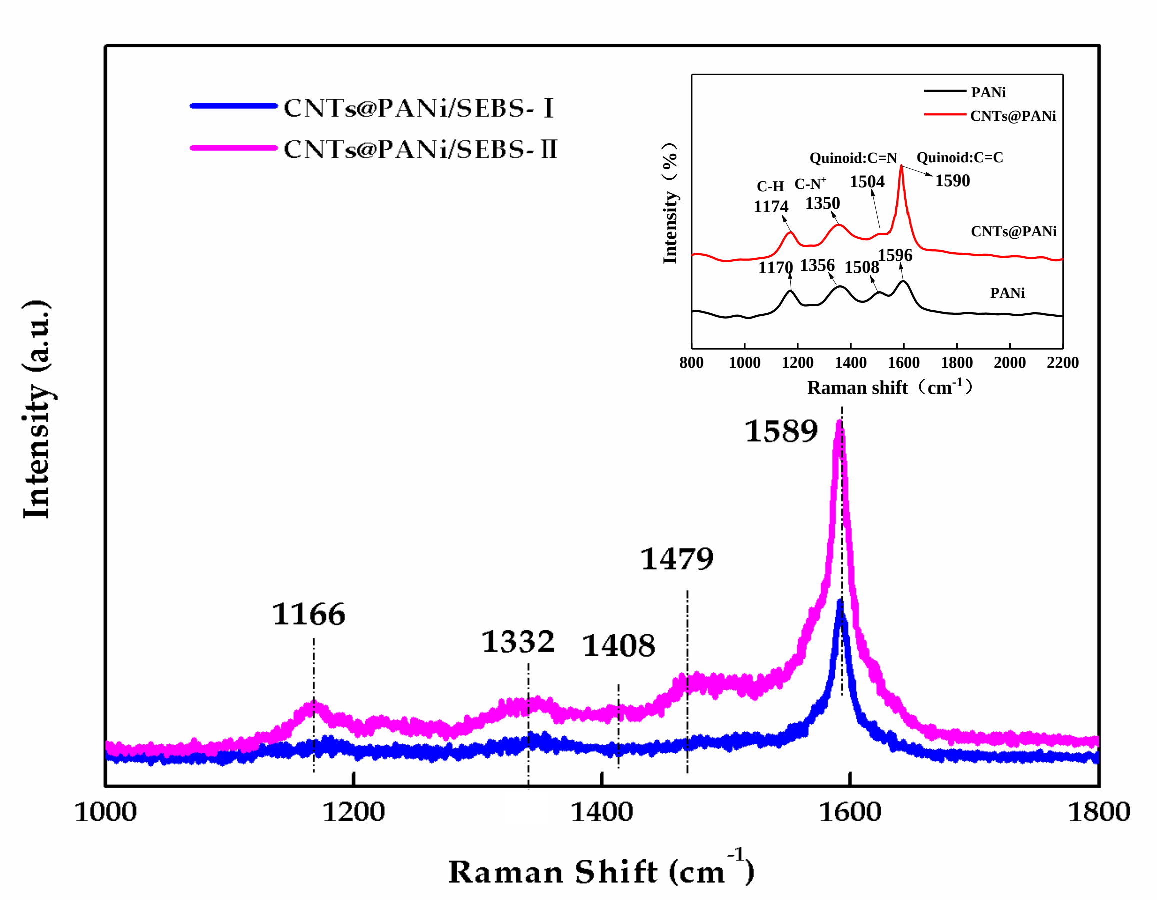

Fig. S5. Raman spectra of CNTs@PANi/SEBS-I and CNTs@PANi/SEBS- II bilayer film CNTs@PANi (function layer), the inset is Raman spectra of PANi and CNTs@PANi.

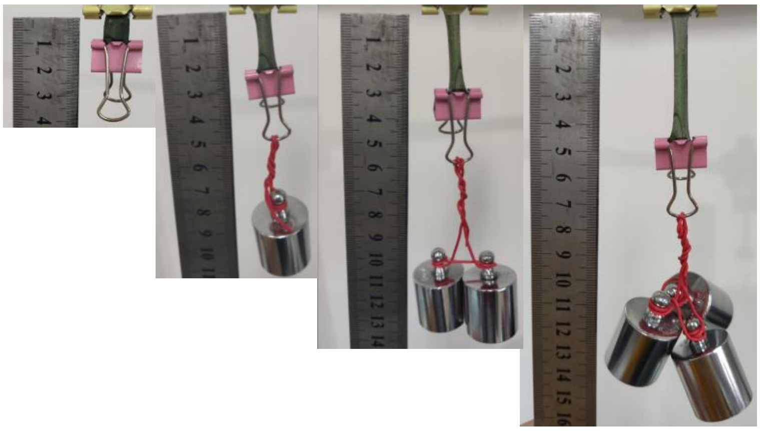

Fig. S6. Stretchable photos of CNTs@PANi/SEBS-I monolayer film.

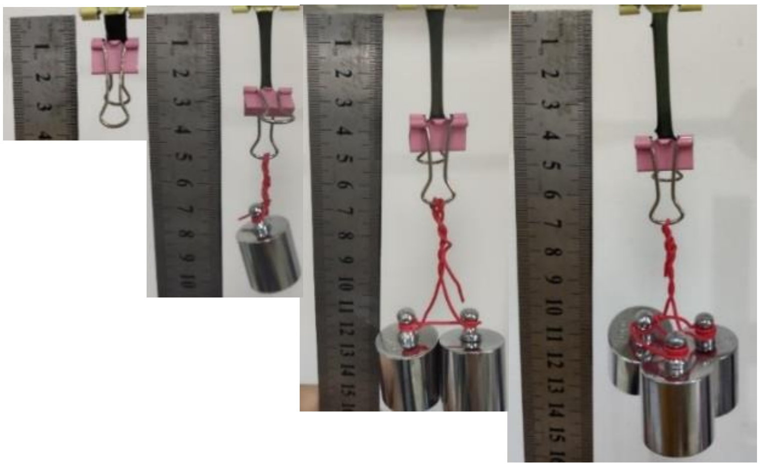

Fig. S7. Stretchable photos of CNTs@PANi/SEBS-Ⅱ bilayer film.

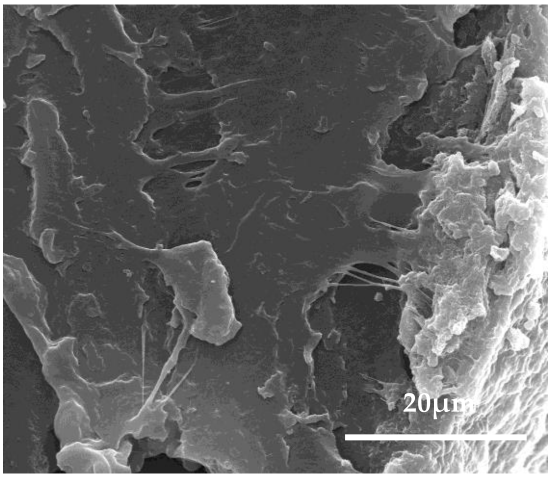

Fig. S8. SEM image of tensile fractured cross-section of CNTs@PANi/SEBS-I monolayer film.

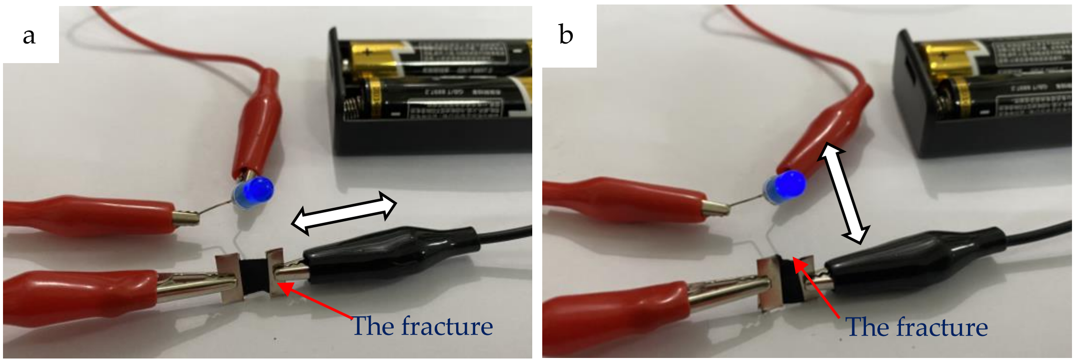

Figure. S9. LED conductivity test of CNTs@PANi/SEBS- II bilayer film after tensile fracture (a) along the stretching direction; (b) perpendicular to the stretching direction.

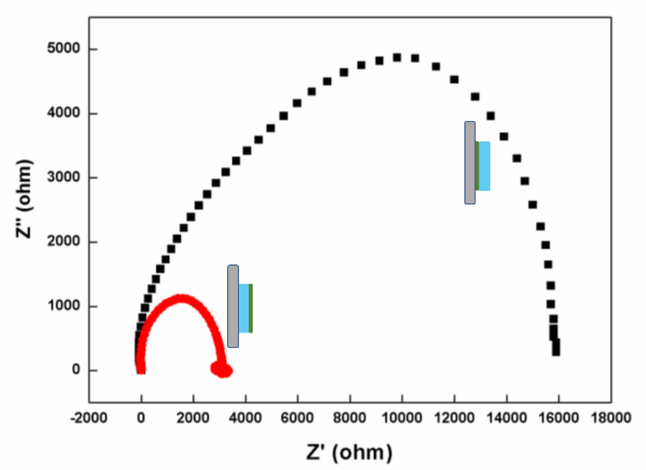

Fig. S10. The impedance curves of the bilayer film with flat surface morphology (doubled CNTs@PANi content in the functional layer).

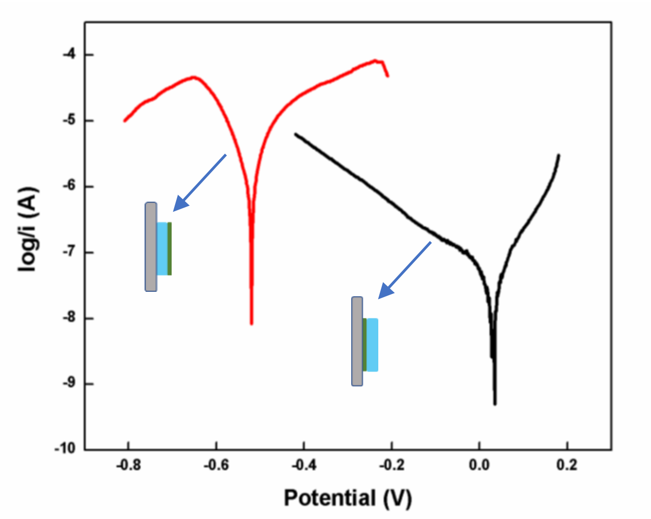

Fig. S11. Tafel curves of the bilayer film with flat surface morphology (doubled CNTs@PANi content in the functional layer).

Table S1. Hildebrand and Hansen's solubility parameters

solvent, polymer	solubility parameters, $MPa^{1/2}$			Hildebrand
	δ_d	δ_p	δ_h	($MPa^{1/2}$)
THF	16.8	5.7	8.0	19.4[1]
Toluene	18.0	1.4	2.0	18.2[1]
SEBS*	-	-	-	17.1[2]
PANi	17.4	8.1	10.7	22.2[3]

* SEBS G1650 (EB/PS=70/30wt%, $M_w=0.7\times10^5$ g mol^{-1}), supplied by Kraton polymers.

Table S2. Physical parameters of different solvent and the Flory–Huggins interaction parameter χ

solvent	vapor pressure(KPa)	boiling point(°C)	dielectric constant	electrical resistivity (MΩ)	χ[1]	χ[2]
					SEBS*	SEBS#
THF	19.3 (20 °C)	66	7.58	2	0.54	0.25
Toluene	4.89 (30 °C)	110.6	2.37	≤20	0.64	0.53

* SEBS G1650 (EB/PS=70/30wt%, $M_w=0.7\times10^5$ g·mol^{-1}), supplied by Kraton polymers.

# SEBS copolymer (EB/PS=68/32wt%, $M_w=0.9\times10^5$ g·mol^{-1}) was synthesized and supplied by Alfonso Cortina Technology Center of REPSOL-YPF.

References

[1] Wu, P.; Qi, S.H.; Liu, N.L.; Deng, K.Q.; Nie, H.Y. Investigation of thermodynamic properties of SIS, SEBS, and naphthenic oil by inverse gas chromatography. *J. Elastom. Plast.* **2011**, *43*, 369-386. https://doi.org/10.1177/0095244311405000

[2] Ovejero, G.; Pérez, P.; Romero, M.D.; Díaz, I.; Díez, E. SEBS triblock copolymer–solvent interaction parameters from inverse gas chromatography measurements. *Eur. Polym. J.* **2009**, *45*, 590-594. https://doi.org/10.1016/j.eurpolymj.2008.11.022

[3] Stewart, K.M.E.; Penlidis, A. Designing polymeric sensing materials: what are we doing wrong? *Polym. Adv. Technol.* **2017**, *28*, 319-344. https://doi.org/10.1002/pat.3893
